# Multi-Region Genomic Landscape Analysis for the Preoperative Prediction of Lymph Node Metastasis in Esophageal Carcinoma

**DOI:** 10.3389/fgene.2022.830601

**Published:** 2022-03-23

**Authors:** Shaofeng Lin, Yanping Chen, Jianchao Wang, Yibin Cai, Xiaohui Chen, Yuanmei Chen, Yi Shi, Gang Chen, Kunshou Zhu

**Affiliations:** ^1^ Department of Thoracic Surgery, Fujian Medical University Cancer Hospital and Fujian Cancer Hospital, Fuzhou, China; ^2^ Department of Pathology, Fujian Medical University Cancer Hospital and Fujian Cancer Hospital, Fuzhou, China; ^3^ Department of Molecular Pathology, Fujian Medical University Cancer Hospital and Fujian Cancer Hospital, Fuzhou, China

**Keywords:** esophageal carcinoma, whole-exome sequencing, lymph node metastases, multi-region sequencing, subclone

## Abstract

**Objective:** Esophageal cancer is an aggressive malignant tumor, with 90 percent of the patients prone to recurrence and metastasis. Although recent studies have identified some potential biomarkers, these biomarkers’ clinical or pathological significance is still unclear. Therefore, it is urgent to further identify and study novel molecular changes occurring in esophageal cancer. It has positive clinical significance to identify a tumor-specific mutation in patients after surgery for an effective intervention to improve the prognosis of patients.

**Methods:** In this study, we performed whole-exome sequencing (WES) on 33 tissue samples from six esophageal cancer patients with lymph node metastasis, compared the differences in the genomic and evolutionary maps in different tissues, and then performed pathway enrichment analysis on non-synonymous mutation genes. Finally, we sorted out the somatic mutation data of all patients to analyze the subclonality of each tumor.

**Results:** There were significant differences in somatic mutations between the metastatic lymph nodes and primary lesions in the six patients. Clustering results of pathway enrichment analysis indicated that the metastatic lymph nodes had certain commonalities. Tumors of the cloned exploration results illustrated that five patients showed substantial heterogeneity.

**Conclusion:** WES technology can be used to explore the differences in regional evolutionary maps, heterogeneity, and detect patients’ tumor-specific mutations. In addition, an in-depth understanding of the ontogeny and phylogeny of tumor heterogeneity can help to further find new molecular changes in esophageal cancer, which can improve the prognosis of EC patients and provide a valuable reference for their diagnosis.

## Introduction

Esophageal carcinoma (EC) is a malignant tumor that occurs in the epithelial tissue of the esophagus. The number of EC patients in China accounts for 70% of EC patients worldwide. EC is highly aggressive and has a poor prognosis, with the 5-year survival rate being only 10–25% (Bolton et al., 2009; Chen et al., 2013; Pennathur et al., 2013; He et al., 2020a). Surgical resection is the primary treatment method for EC (Chadwick et al., 2016). However, 90% of EC patients who underwent surgery had recurrence and metastasis. Nearly half of them recurred within 5 years after surgery (Koshy et al., 2004; Scheithauer, 2004), and the annual survival rate was only about 40% (Allum et al., 2011; Alderson et al., 2015). After surgery, the local recurrence and distant metastasis are still clinically challenging issues for most cancers (He et al., 2020b; Liu et al., 2021). Many factors influence the prognosis of EC patients undergoing radical resection, such as living environment, clinicopathological features, molecular biological indicators, and treatment methods. It has been reported that DNA damage and repair processes lead to somatic mutations in cancer genomes (Pleasance et al., 2010). Recent studies have identified some genomic abnormalities as potential biomarkers for EC (Guo et al., 2013; Zhu et al., 2013), but these biomarkers’ clinical or pathological significance remains unclear. Therefore, further studies are urgently needed to identify new molecular changes in EC.

It is well known that genetic changes are the root cause of tumorigenesis, and cancers are caused by the accumulation of genomic alterations (Meyerson et al., 2010). With the progress of sequencing technology (next-generation sequencing, NGS), more and more studies are using whole-exome sequencing (WES) to study the comprehensive molecular characteristics of cancer, which allows querying thousands of variants of multiple genes in a given tumor sample at the same time (Ng et al., 2010; The Cancer Genome Atlas Network, 2012). Recent cancer genome analysis compared multiple samples of a single individual to gain insights into the evolutionary history of the cancer genome (Meyerson et al., 2010). For example, the primary tumor genome was compared with the matched metastatic tumor genome (Yachida et al., 2010). Based on WES technology, many driving genes and several critical signaling pathways of EC pathogenesis have been identified (Dulak et al., 2013; Zhang et al., 2015; Cheng et al., 2016; Deng et al., 2017; The Cancer Genome Atlas Research Network, 2017). However, the biological relationship between different intratumoral clonal subgroups is still unclear. Some scholars have begun to explore the diagnosis and treatment of tumors from the perspective of tumor heterogeneity (Liu et al., 2016; Furuta et al., 2017; Wang et al., 2020). A study published in 2015 conducted regional segmentation of the same lesion in patients with ESCC and used WES to explore tumor heterogeneity (Cao et al., 2015). Che et al. used WES to examine mutational concordance and heterogeneity between EC patients with matched dysplasia and carcinomatosis and tumor-free patients with only dysplasia samples. By performing clonal evolutionary analysis of individual patients, it has been found that most driver mutations of EC are also present in dysplastic tissue (Chen et al., 2017). All the aforementioned studies have shown that tumors evolve through different subclones, so there is heterogeneity among tumors, which leads to tumor recurrence and drug resistance (Gerlinger et al., 2012; Fisher et al., 2013; Sottoriva et al., 2013). Therefore, detection of tumor-specific mutations in patients with EC after surgery is helpful to timely take effective intervention measures, which is an effective way to improve the survival rate of EC patients and has positive clinical significance to improve their prognosis.

In this study, WES was applied to lymph node (LN) metastasis samples of EC patients to compare the difference of genomic landscape and evolution map in multiregional LNs and explore tumor heterogeneity based on somatic mutation information.

## Materials and Methods

### Sample Collection

To explore the heterogeneity of EC, we collected the information of esophageal cancer patients from Fujian Cancer Hospital. Patients included were with the following criteria: male patients undergone surgery; patients with corresponding case data and histological specimens; and patients diagnosed with LN metastasis. Written informed consent was obtained from all participants. A total of 33 tumor tissue samples and lymph node metastasis samples were obtained from 6 EC patients. These samples included seven primary carcinomas, 20 LNs with metastatic carcinoma, and six normal mucosae (Table1). The detailed pathological information and clinical information (Table 2), such as the prognosis of the enrolled patients, were collected simultaneously. WES was performed with DNA isolated from tissues.

**TABLE 1:** Information of all samples in six patients.

Case 1	Site	Sample type	Sample number
Patient 1	2R lymph nodes	FFPE	P1_2R
	Cardia lymph nodes	FFPE	P1_cardia
	17 lymph nodes	Frozen tissue	P1_17
	Normal mucosa	FFPE	P1_normal
	Primary esophageal cancer	FFPE	P1_primary
Patient 2	2R lymph nodes	FFPE	P2_2R
	16 lymph nodes	FFPE	P2_16
	7 lymph nodes	FFPE	P2_7
	Cardia lymph node	FFPE	P2_ cardia
	1 L cardia lymph node	Frozen tissue	P2_1 L
	Normal mucosa	FFPE	P2_normal
	110 lymph nodes	FFPE	P2_110
	Primary esophageal cancer	FFPE	P2_primary
Patient 3	Left parapharyngeal lymph node	FFPE	P3_left
	7 lymph nodes	FFPE	P3_7
	Primary esophageal cancer	Frozen tissue	P3_primary
	1 L lymph nodes	Frozen tissue	P3_1 L
	Normal mucosa	FFPE	P3_normal
Patient 4	Right parapharyngeal lymph node	FFPE	P4_right
	7 lymph nodes	FFPE	P4_7
	Normal mucosa	FFPE	P4_normal
	Left parapharyngeal lymph node	FFPE	P4_left
	Primary esophageal cancer	Frozen tissue	P4_primary
	17 lymph nodes	Frozen tissue	P4_17
Patient 5	Cardia lymph node	FFPE	P5_ cardia
	2R cardia lymph node	Frozen tissue	P5_2R
	Normal mucosa	FFPE	P5_normal
	Primary esophageal cancer	FFPE	P5_primary
Patient 6	8M cardia lymph node	FFPE	P6_8M
	Primary esophageal cancer	Frozen tissue	P6_primary1
	Left parapharyngeal lymph node	Frozen tissue	P6_left
	Normal mucosa	FFPE	P6_normal
	Primary esophageal cancer	FFPE	P6_primary2

**TABLE 2:** Summary of the general clinical information of EC patients.

Patient	Gender	Age (years)	TNM stage	T	N	M	Survival	Smoking
Patient 1	Male	70	ⅢC	3	3	0	Dead	No
Patient 2	Male	47	ⅢC	4	2	0	Dead	Yes
Patient 3	Male	54	ⅢA	3	2	0	Alive	Yes
Patient 4	Male	56	ⅢB	3	2	0	Alive	Yes
Patient 5	Male	65	ⅢA	3	1	0	Unknown	Yes
Patient 6	Male	76	ⅢA	3	1	0	Dead	No

### DNA Extraction and Quantification

Genomic DNA from fresh tumors and normal tissue were extracted using a DNeasy Blood & Tissue Kit (Qiagen). Purified genomic DNA was qualified by using Nanodrop2000 for A260/280 and A260/A230 ratios (Thermo Fisher Scientific). According to the manufacturer’s recommendations, all DNA samples were quantified by using Qubit 3.0 and a dsDNA HS Assay Kit (Life Technologies). Genomic DNA from normal lung tissue was used as the normal control.

### Library Preparation

Sequencing libraries were prepared using the KAPA Hyper Prep kit (KAPA Biosystems) with an optimized manufacturer’s protocol. Briefly, 1 μg of genomic DNA was sheared into 350-bp fragments using a Covaris M220 instrument (Covaris), followed by end repair, A-tailing, and ligation with index sequencing adapters. Then, size selection for genomic DNA libraries was carried out using Agencourt AMPure XP beads (Beckman Coulter). Finally, libraries were amplified by using PCR and purified using Agencourt AMPure XP beads.

### Exome Sequencing and Data Processing

Exome capture was performed using the IDT xGen Exome Research Panel V1.0 (Integrated DNA Technologies). Enriched libraries were sequenced using the Illumina HiSeq 4000 platform to reach the mean coverage depth of ∼60X for the normal control (normal lung tissue) and ∼200X for the tumor samples.

Paired-end sequencing reads were aligned to the reference human genome (build hg19) with the Burrows–Wheeler Aligner (bwa-mem). Alignment results (BAM files) were further processed for de-duplication, base quality recalibration, and indel realignment using the Picard suite (http://picard.sourceforge.net/) and the Genome Analysis Toolkit (GATK). MuTect with default parameters was applied to paired normal and tumor BAM files for the identification of somatic single-nucleotide variants (SNVs). SNVs in the 1000 Genomes Project and dbSNP with a frequency > 1% were excluded. Small insertions and deletions (indels) were detected using SCALPEL. SNV and indel annotations were performed by ANNOVAR using the reference genome hg19 and standard databases version 2014 and functional prediction programs. Gene-level copy number ratios were calculated by CNVKit. Relative copy-ratios for each exon were calculated by correcting for imbalanced library size, GC bias, sequence repeats, and target density using the CNVKit algorithm.

### Pathway Enrichment Analysis

We conducted KEGG enrichment for non-synonymous mutation genes to extend significance analysis beyond individual genes. We checked the distribution of non-synonymous mutation genes identified in KEGG and performed pathway enrichment analysis, labeling, and visualization in the database by observing the genome.

### Tumor Heterogeneity Analysis

PyClone was used to conduct statistical analysis on somatic mutation data (Roth et al., 2014). PyClone is a stratified Bayesian model that inferred the cell prevalence for each variant (the percentage of tumor cells in the sample containing the variant), clustering the variants based on the covariance of multiple sample prevalence estimates for the same patient (Findlay et al., 2016; Lamy et al., 2016; McPherson et al., 2016). We used Citup for evolutionary analysis to infer the subclonal of EC patients. Citup, a bioinformatics tool for tumor clonal inference using phylogenetic theory, can infer tumor heterogeneity from multiple samples obtained from a single patient (Malikic et al., 2015). Given the mutation frequency of each sample, Citup uses an optimization-based algorithm to find the evolutionary tree that can best explain the data and infer the tumor clone with phylogeny. This method can use data from multiple samples to deduce clonal populations and their frequencies subject to phylogenetic constraints. To sum up, mutation data from multiple samples of the same patient were used as the input data to software PyClone. Then, allele-specific copy number measurements were made for each mutation site in each sample. Second, evolutionary analyses were performed using Citup. Finally, the results were visualized with the R package *timescape*.

## Results

### Framework for This Study

**FIGURE 1:**
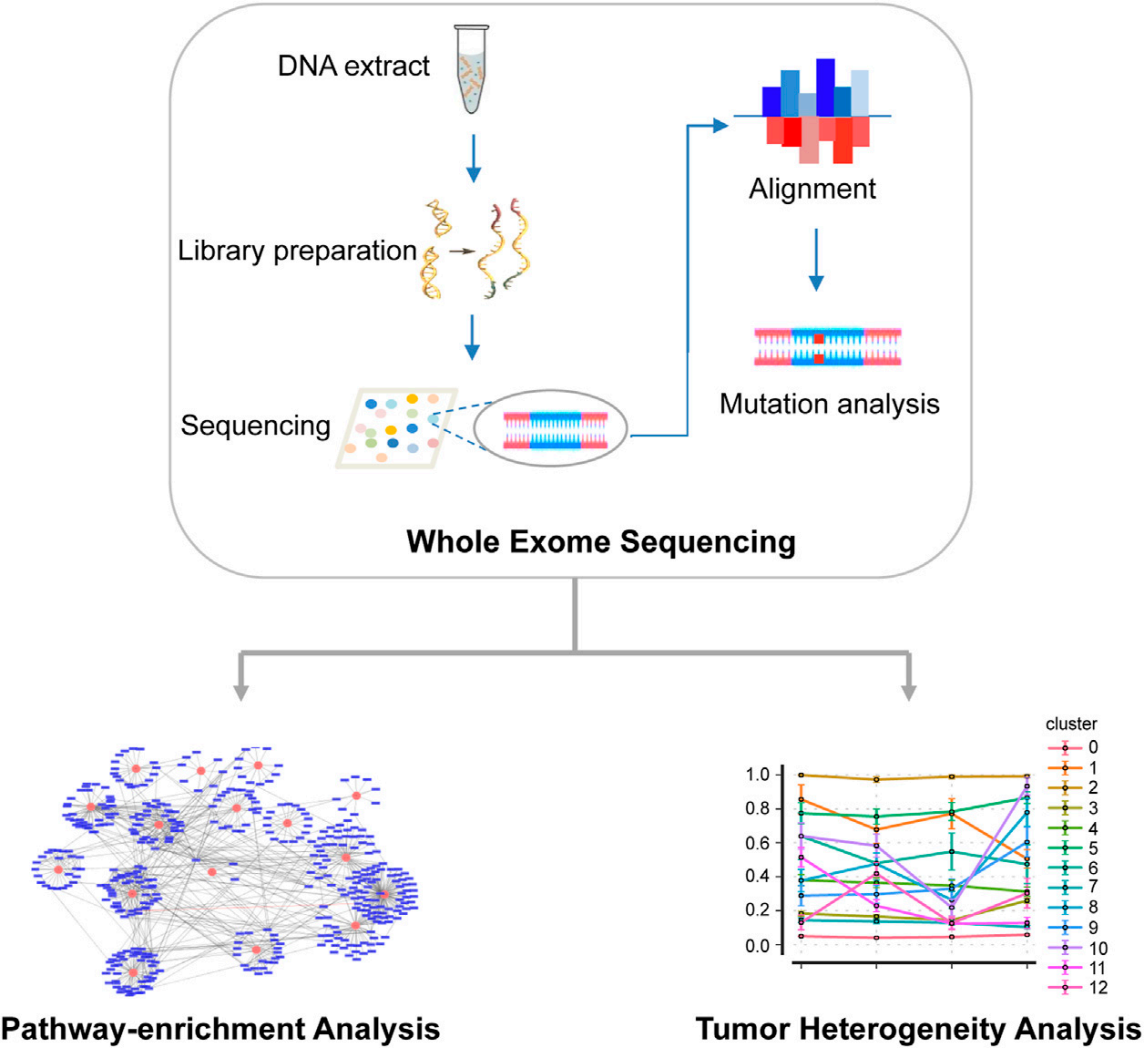
Overview of the main workflow.

An overview of the main workflow for this study is shown in Figure 1. Specifically, after DNA was extracted from tumor and normal tissues, a gene library was prepared and sequenced using Illumina HiSeq 4000 platform. The sequencing reads were then compared with the reference human genome to identify the somatic SNV. Based on the results of mutation analysis, KEGG was used for pathway enrichment analysis of non-synonymous mutations. In addition, somatic mutation data were statistically analyzed to infer subclones of EC patients.

### There Are Differences in Somatic Mutations Among Primary Tumors and Metastatic LNs

**FIGURE 2:**
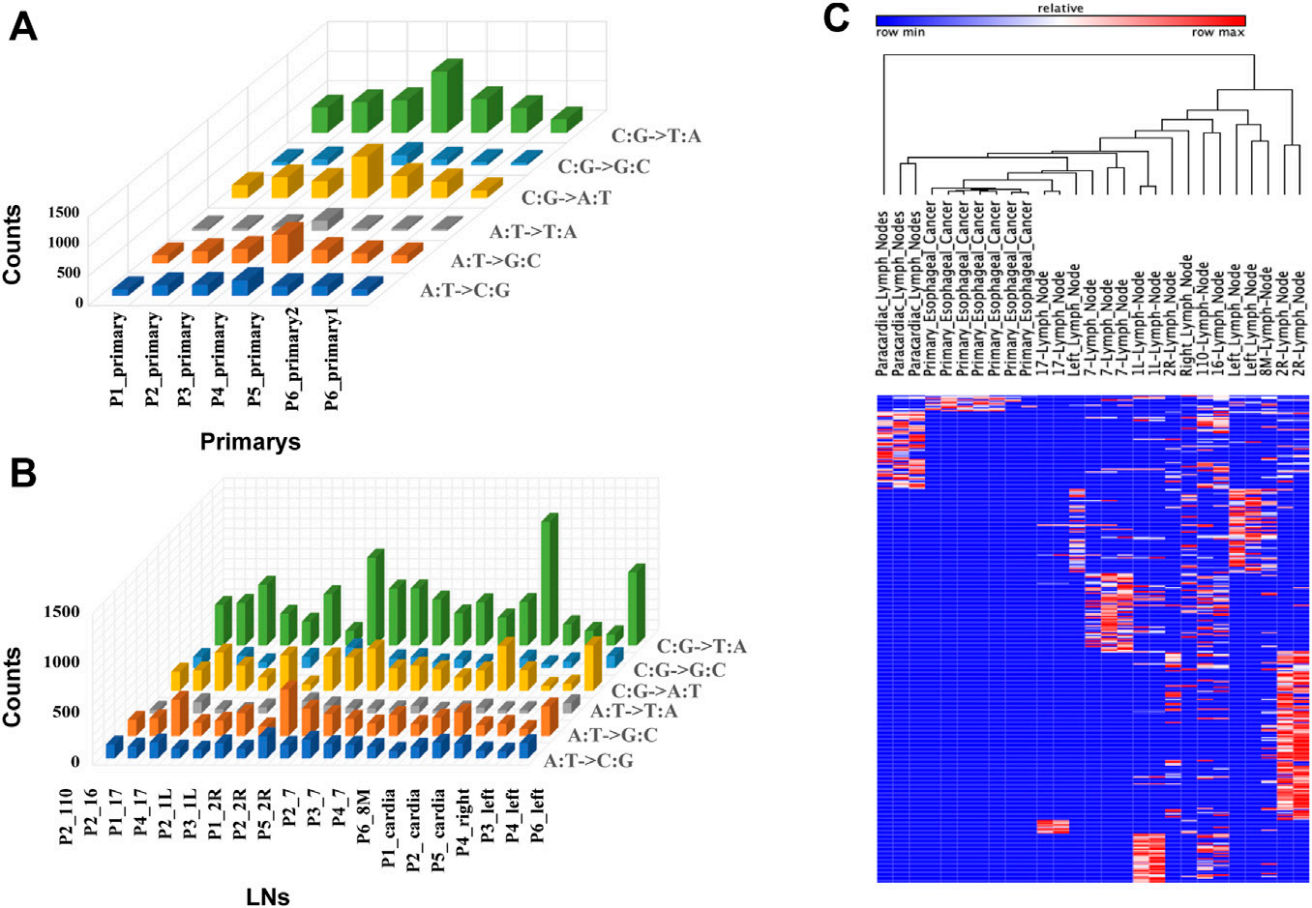
Somatic mutations in tumor samples. **(A)** Histogram of non-synonymous mutation counts with mutation frequency greater than 5% in primary samples. **(B)** Histogram of non-synonymous mutation counts with mutation frequency greater than 5% in LN samples. **(C)** Mutation spectrum of LNs and primary: the column represents the sample, the row represents the site, and each cell represents the mutation count of the sample at that site.

Thirty-three formalin-fixed paraffin-embedded tissues (FFPE) and frozen tissue samples from six EC patients were collected. WES was performed on all samples to further explore potential heterogeneity and the cloning progress, and SamTools was applied to invoke somatic variations. To identify specific mutations at this locus, we screened non-identical and frameshift mutations with a mutation frequency greater than 5%. For all primary sites and metastatic LNs, the total number of mutations (non-homozygous and frame-coding mutations with a mutation frequency of not less than 5%) ranged from 18 to 1,221. Specific mutations are shown in Figures 2A,B. We observed that C: G > T: A had the largest number of mutations both in metastatic LNs and primary lesions. For primary lesions of all patients, the largest number of C: G > T: A occurred in patient 5. The heatmap in Figure 2C shows all the metastatic LNs and primary mutations at each site. It can be seen that the mutations of metastatic LNs and primary were distinct, indicating that there are differences in somatic mutations between metastatic LNs and primary lesions.

### Metastatic LNs Have Pathway Commonality

**FIGURE 3:**
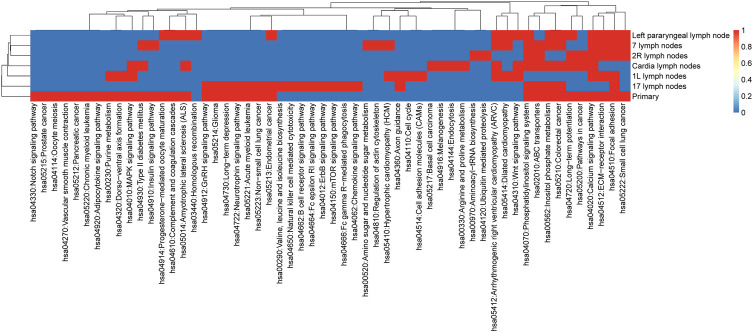
KEGG pathway enrichment in multi-region of primary and metastatic LNs.

KEGG enrichment analysis for non-synonymous mutant genes was used to extend the significance analysis beyond a single gene. Our results (shown in Figure 3) indicated that metastatic LNs had certain commonalities. Lymph nodes and primary foci shared many pathways, such as hsa04512: ECM receiver interaction, hsa00562: inositol phosphate metabolism, hsa05210: colorectal cancer, hsa04070: phosphatidylinositol signaling system, hsa04020: calcium signaling pathway, and hsa04510: focal adhesion. These signaling pathways are often dysregulated in a variety of cancers, which enhances confidence in the study of their respective mechanisms.

The pathways of primary focus acting alone were hsa04330: Notch signaling pathway, hsa05215: prostate cancer, hsa04114: oocyte meiosis, hsa04270: vascular smooth muscle contraction, hsa05212: pancreatic cancer, hsa05220: chronic myeloid leukemia, and hsa04920: adipocytokine signaling pathway. The Notch signaling pathway mediates different biological processes, including stem cell self-renewal, progenitor cell fate determination, and terminal differentiation. The expression of the Notch pathway core transcription complex and its target genes was closely related to the invasive clinicopathological variables of esophageal squamous cell carcinoma (ESCC). In conclusion, this result suggests that the normal function of the aforementioned signal pathways may be widely affected by the related mutant genes and is helpful to the development of heterogeneity research of EC.

### Primary and LNs Are Heterogeneous

**FIGURE 4:**
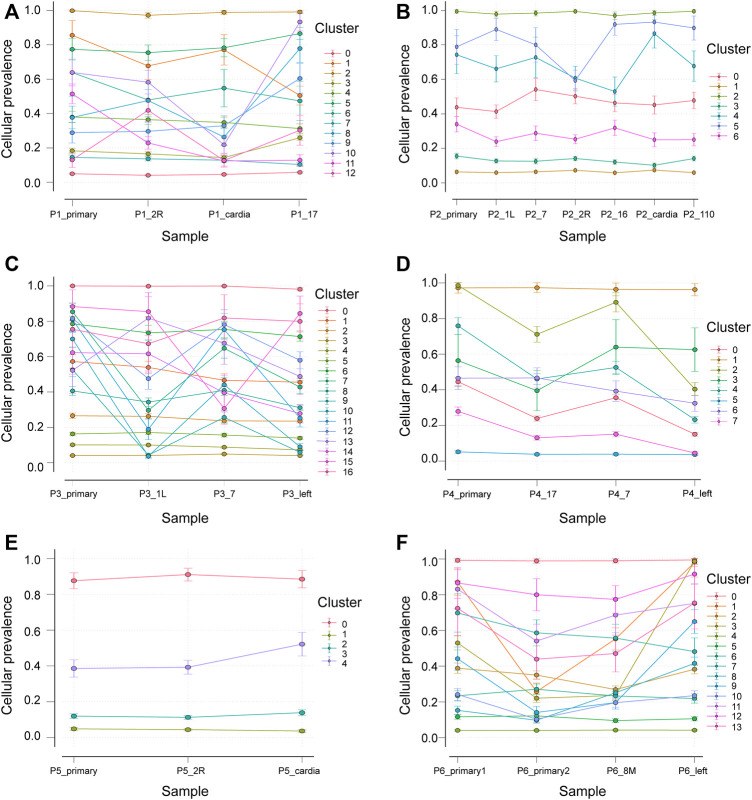
Subclones of primary and metastatic LNs in all samples generated by using PyClone were composed. Each panel represents a patient, the *x*-axis represents not a time but a sample, and the *y*-axis represents the mean cell prevalence of variation in all clusters in each sample. **(A–F)** Patients 1 to 6.

We collated somatic mutation data from all patients to analyze and explore the evolutionary relationship between primary and metastases LNs at the molecular level. We analyzed the subclonality of each tumor using PyClone. The cell prevalence of each variant was estimated using the copy number and tumor purity based on its allele frequency. The results of different clustering types according to the cell prevalence distribution of different samples are shown in Figure 4. It is important to note that the sample order is not chronological but that primary and metastatic LNs were collected simultaneously. The results showed that different patients had different subclones. Even for the same patient, the prevalence of cells in distinct clusters varied in different samples. For example, subclone 10 from patient 1 (Figure 4A) has a low cellular prevalence in cardiac lymph nodes but is higher in other samples. The other cluster 11 of patient 1 was almost exclusively present in the primary lymph nodes, with little or no presence in other samples. Clusters 9 and 10 in patient 3 (Figure 4C) were found in primary and seven lymph nodes. Similarly, patient 6 also showed substantial heterogeneity (Figure 4F). Interestingly, according to the survival information in Table 2, both patient 1 and patient 6 died, suggesting that tumor heterogeneity may affect the prognosis and survival of patients.

**FIGURE 5:**
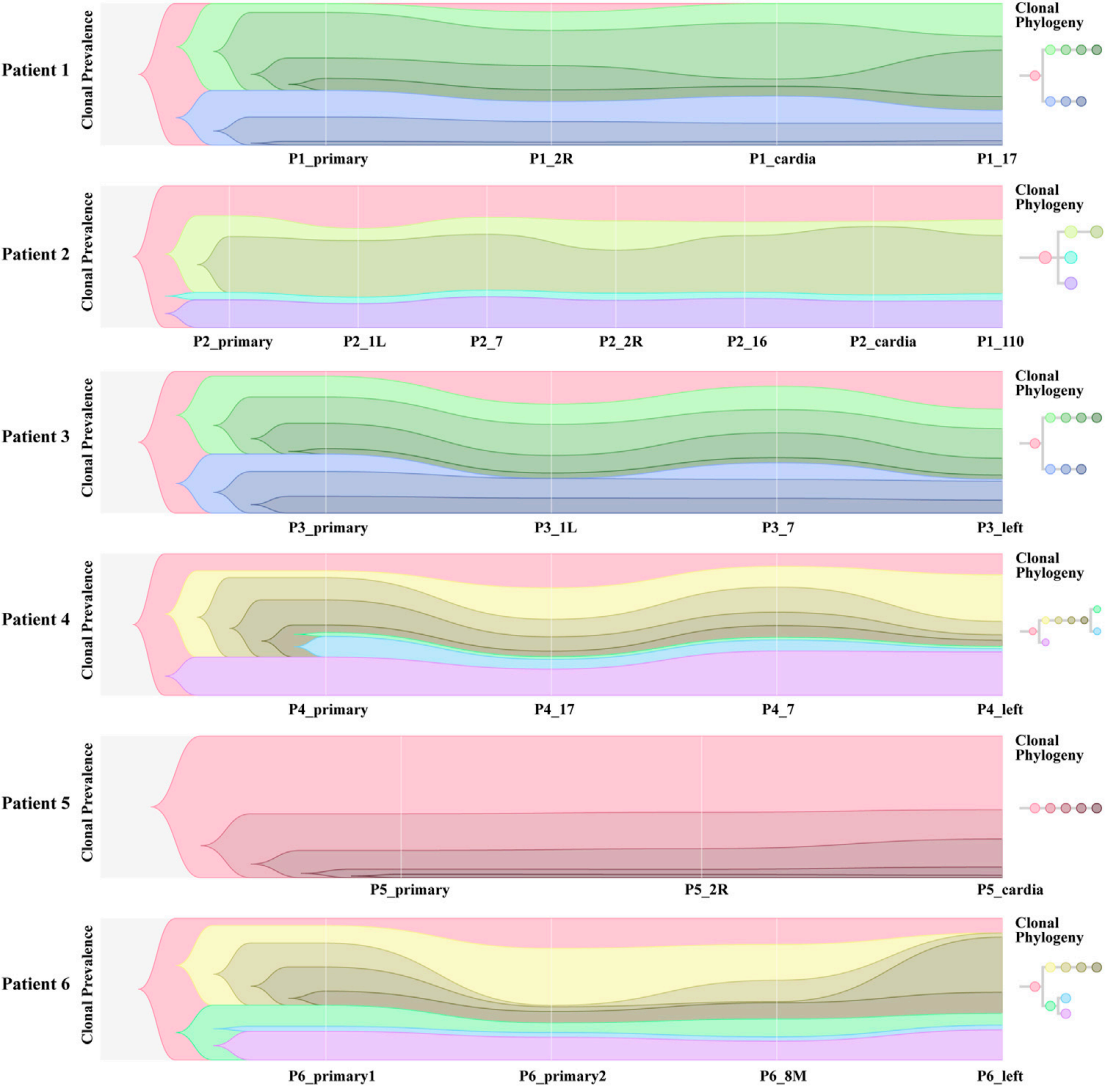
Fish diagram of each patient’s tumor clone envelogram.

To further investigate the inference of tumor clones, we conducted an evolutionary analysis of mutant clusters and cell prevalence in all samples to identify the parent clones of each tumor clone. According to the analysis results of Citup, the metastatic evolution diagrams of all patients are shown in Figure 5. The results showed that the founder clone of the primary tumor was absent. However, the most persistent clones were traced back to the primary tumor during disease progression. Except for patient 5, the samples of all patients showed significant heterogeneity. For patient 6, in particular, the clones not present in primary 2 were present in the left lymph node, while clones present in primary 2 were not in the left lymph node. This indicated a substantial heterogeneity between primary 2 and left lymph nodes. The distribution of these subclones in different sites of all samples probably reflected the prognosis of patients.

## Discussion

Esophageal cancer is highly invasive and prone to recurrence and metastasis. Recent whole-exome sequencing and whole-genome sequencing have shown that patients with EC have a high mutation rate (Song et al., 2014; Zhang et al., 2015), with a large number of copy number changes and large-scale chromosome rearrangements. Hence, it is urgent to further study new molecular changes in EC. With the rapid development of cancer genome sequencing, the genomic heterogeneity between and within tumors has been a vital cancer feature (Zhang et al., 2013; Murugaesu et al., 2015). It is of positive significance to detect whether there are tumor-specific mutations in patients and take effective intervention measures to improve patients’ prognosis and survival rate.

This study collected 33 samples from six EC patients, including tumor tissue and lymph node metastases. WES was performed on these samples to compare genomic and evolutionary maps in different regions. Then, we performed pathway enrichment on non-synonymous mutation genes. Finally, the subclonality of each tumor based on the somatic mutation data of all patients was analyzed. Results showed that the total number of mutations in metastatic LNs and primary ranged from 18 to 1,221. The largest number of mutations found was C: G > T: A. There were significant differences in somatic mutations between metastatic lymph nodes and primary lesions in six patients. Moreover, the metastatic LNs had certain commonalities based on the clustering results of pathway enrichment. For example, ECM–receptor interaction is the most abundant signal pathway in the ESCC cell line (Ma et al., 2021). The extracellular matrix (ECM) serves an essential role in tissue and organ morphogenesis and maintains cell and tissue structure and function. Transmembrane molecules mediate specific interactions between cells and the ECM. These interactions lead to direct or indirect control of cellular activities such as adhesion, migration, differentiation, proliferation, and apoptosis. But the primary had some independent pathways. Notch and other developmental pathways are involved in different cell functions from cell cycle regulation to self-renewal (Moghbeli et al., 2015). The expression of Notch pathway core transcription complex and its target gene and the overexpression of *TWIST1* are closely related to the invasive clinicopathological variables of ESCC (Fahim et al., 2020). Adipokines play a significant regulatory role in the adipocytokine signaling pathway. The increase in the adipocyte volume and number is positively correlated with leptin production and negatively associated with adiponectin production. Obesity may increase the risk of ESCC and affect its growth and progression (Liu et al., 2020). Tumors of the cloned exploration results showed that different patients had distinct subclones. Even for the same patient, the prevalence of cells in different clusters varied among samples.

It is of notice that there are a few limitations to this study. First, we only focused on genomic changes, and other levels of molecular data such as gene expression have also been proven vital in studying the recurrence and metastasis of many cancers (He et al., 2020c). Second, since EC is quite heterogeneous, it might be helpful to check EC samples at the single-cell level to identify cell clusters (Xu et al., 2020; Zhuang et al., 2021). Finally, the number of patients was limited, and the increase in the sample size might provide more meaningful findings.

In summary, we used WES to explore the differences in the evolution map and heterogeneity in different regions and detect tumor-specific mutations in patients to help improve the prognosis of EC patients. However, there are still some limitations to this study. The original data are insufficient, which is an urgent problem to be solved. In the future, we will intake more cases of EC, increase the sample size, and deeply understand the ontogeny and phylogeny of tumor heterogeneity to further identify new molecular changes in EC.

## Data Availability

The original contributions presented in the study are publicly available. This data can be found here: PRJNA791155.

## References

[B1] Alderson D. Langley R. E. Nankivell M. G. Blazeby J. M. Griffin M. Crellin A. (2015). Neoadjuvant Chemotherapy for Resectable Oesophageal and Junctional Adenocarcinoma: Results from the UK Medical Research Council Randomised OEO5 Trial (ISRCTN 01852072). J. Clin. Oncol. 33 (15_Suppl. l), 4002. 10.1200/jco.2015.33.15_suppl.4002

[B2] Allum W. H. Blazeby J. M. Griffin S. M. Cunningham D. Jankowski J. A. Wong R. (2011). Guidelines for the Management of Oesophageal and Gastric Cancer. Gut 60 (11), 1449–1472. 10.1136/gut.2010.228254 21705456

[B3] Bolton W. D. Hofstetter W. L. Francis A. M. Correa A. M. Ajani J. A. Bhutani M. S. (2009). Impact of Tumor Length on Long-Term Survival of pT1 Esophageal Adenocarcinoma. J. Thorac. Cardiovasc. Surg. 138 (4), 831–836. 10.1016/j.jtcvs.2009.02.003 19660349

[B4] Cao W. Wu W. Yan M. Tian F. Ma C. Zhang Q. (2015). Multiple Region Whole-Exome Sequencing Reveals Dramatically Evolving Intratumor Genomic Heterogeneity in Esophageal Squamous Cell Carcinoma. Oncogenesis 4 (11), e175. 10.1038/oncsis.2015.34 26619400 PMC4670960

[B5] Chadwick G. Groene O. Taylor A. Riley S. Hardwick R. H. Crosby T. (2016). Management of Barrett's High-Grade Dysplasia: Initial Results From a Population-Based National Audit. Gastrointest Endosc. 83 (4), 736–742.e1. 10.1016/j.gie.2015.08.020 26283273

[B6] Chen M.-F. Yang Y.-H. Lai C.-H. Chen P.-C. Chen W.-C. (2013). Outcome of Patients with Esophageal Cancer: A Nationwide Analysis. Ann. Surg. Oncol. 20 (9), 3023–3030. 10.1245/s10434-013-2935-4 23525703

[B7] Chen X.-X. Zhong Q. Liu Y. Yan S.-M. Chen Z.-H. Jin S.-Z. (2017). Genomic Comparison of Esophageal Squamous Cell Carcinoma and its Precursor Lesions by Multi-Region Whole-Exome Sequencing. Nat. Commun. 8 (1), 524. 10.1038/s41467-017-00650-0 28900112 PMC5595870

[B8] Cheng C. Zhou Y. Li H. Xiong T. Li S. Bi Y. (2016). Whole-Genome Sequencing Reveals Diverse Models of Structural Variations in Esophageal Squamous Cell Carcinoma. Am. J. Hum. Genet. 98 (2), 256–274. 10.1016/j.ajhg.2015.12.013 26833333 PMC4746371

[B9] Deng J. Chen H. Zhou D. Zhang J. Chen Y. Liu Q. (2017). Comparative Genomic Analysis of Esophageal Squamous Cell Carcinoma between Asian and Caucasian Patient Populations. Nat. Commun. 8 (1), 1533. 10.1038/s41467-017-01730-x 29142225 PMC5688099

[B10] Dulak A. M. Stojanov P. Peng S. Lawrence M. S. Fox C. Stewart C. (2013). Exome and Whole-Genome Sequencing of Esophageal Adenocarcinoma Identifies Recurrent Driver Events and Mutational Complexity. Nat. Genet. 45 (5), 478–486. 10.1038/ng.2591 23525077 PMC3678719

[B11] Fahim Y. Yousefi M. Izadpanah M. H. Forghanifard M. M. (2020). TWIST1 Correlates with Notch Signaling Pathway to Develop Esophageal Squamous Cell Carcinoma. Mol. Cel Biochem 474 (1-2), 181–188. 10.1007/s11010-020-03843-2 32712748

[B12] Findlay J. M. Castro-Giner F. Makino S. Rayner E. Kartsonaki C. Cross W. (2016). Differential Clonal Evolution in Oesophageal Cancers in Response to Neo-Adjuvant Chemotherapy. Nat. Commun. 7, 11111. 10.1038/ncomms11111 27045317 PMC4822033

[B13] Fisher R. Pusztai L. Swanton C. (2013). Cancer Heterogeneity: Implications for Targeted Therapeutics. Br. J. Cancer 108 (3), 479–485. 10.1038/bjc.2012.581 23299535 PMC3593543

[B14] Furuta M. Ueno M. Fujimoto A. Hayami S. Yasukawa S. Kojima F. (2017). Whole Genome Sequencing Discriminates Hepatocellular Carcinoma with Intrahepatic Metastasis from Multi-Centric Tumors. J. Hepatol. 66 (2), 363–373. 10.1016/j.jhep.2016.09.021 27742377

[B15] Gerlinger M. Rowan A. J. Horswell S. Math M. Larkin J. Endesfelder D. (2012). Intratumor Heterogeneity and Branched Evolution Revealed by Multiregion Sequencing. N. Engl. J. Med. 366 (10), 883–892. 10.1056/NEJMoa1113205 22397650 PMC4878653

[B16] Guo W. Zhu T. Dong Z. Cui L. Zhang M. Kuang G. (2013). Decreased Expression and Aberrant Methylation of Gadd45G Is Associated with Tumor Progression and Poor Prognosis in Esophageal Squamous Cell Carcinoma. Clin. Exp. Metastasis 30 (8), 977–992. 10.1007/s10585-013-9597-2 23793925

[B17] He B. Dai C. Lang J. Bing P. Tian G. Wang B. (2020). A Machine Learning Framework to Trace Tumor Tissue-Of-Origin of 13 Types of Cancer Based on DNA Somatic Mutation. Biochim. Biophys. Acta (Bba) - Mol. Basis Dis. 1866 (11), 165916. 10.1016/j.bbadis.2020.165916 32771416

[B18] He B. Lang J. Wang B. Liu X. Lu Q. He J. (2020). TOOme: A Novel Computational Framework to Infer Cancer Tissue-Of-Origin by Integrating Both Gene Mutation and Expression. Front. Bioeng. Biotechnol. 8, 394. 10.3389/fbioe.2020.00394 32509741 PMC7248358

[B19] He B. Zhang Y. Zhou Z. Wang B. Liang Y. Lang J. (2020). A Neural Network Framework for Predicting the Tissue-Of-Origin of 15 Common Cancer Types Based on RNA-Seq Data. Front. Bioeng. Biotechnol. 8, 737. 10.3389/fbioe.2020.00737 32850691 PMC7419649

[B20] Koshy M. Esiashvilli N. Landry J. C. Thomas C. R. Matthews R. H. (2004). Multiple Management Modalities in Esophageal Cancer: Combined Modality Management Approaches. Oncologist 9 (2), 147–159. 10.1634/theoncologist.9-2-147 15047919

[B21] Lamy P. Nordentoft I. Birkenkamp-Demtröder K. Thomsen M. B. H. Villesen P. Vang S. (2016). Paired Exome Analysis Reveals Clonal Evolution and Potential Therapeutic Targets in Urothelial Carcinoma. Cancer Res. 76 (19), 5894–5906. 10.1158/0008-5472.CAN-16-0436 27488526

[B22] Liu H. Qiu C. Wang B. Bing P. Tian G. Zhang X. (2021). Evaluating DNA Methylation, Gene Expression, Somatic Mutation, and Their Combinations in Inferring Tumor Tissue-Of-Origin. Front. Cel Dev. Biol. 9, 619330. 10.3389/fcell.2021.619330 PMC8126648 34012960

[B23] Liu J. H. Wu Q. F. Fu J. K. Che X. M. Li H. J. (2020). Obesity Potentiates Esophageal Squamous Cell Carcinoma Growth and Invasion by AMPK-YAP Pathway. J. Immunol. Res. 2020, 6765474. 10.1155/2020/6765474 33381605 PMC7748896

[B24] Liu Y. Zhang J. Li L. Yin G. Zhang J. Zheng S. (2016). Genomic Heterogeneity of Multiple Synchronous Lung Cancer. Nat. Commun. 7, 13200. 10.1038/ncomms13200 27767028 PMC5078731

[B25] Ma F. Laster K. Nie W. Liu F. Kim D. J. Lee M.-H. (2021). Heterogeneity Analysis of Esophageal Squamous Cell Carcinoma in Cell Lines, Tumor Tissues and Patient-Derived Xenografts. J. Cancer 12 (13), 3930–3944. 10.7150/jca.52286 34093800 PMC8176252

[B26] Malikic S. McPherson A. W. Donmez N. Sahinalp C. S. (2015). Clonality Inference in Multiple Tumor Samples Using Phylogeny. Bioinformatics 31 (9), 1349–1356. 10.1093/bioinformatics/btv003 25568283

[B27] McPherson A. Roth A. Laks E. Masud T. Bashashati A. Zhang A. W. (2016). Divergent Modes of Clonal Spread and Intraperitoneal Mixing in High-Grade Serous Ovarian Cancer. Nat. Genet. 48 (7), 758–767. 10.1038/ng.3573 27182968

[B28] Meyerson M. Gabriel S. Getz G. (2010). Advances in Understanding Cancer Genomes through Second-Generation Sequencing. Nat. Rev. Genet. 11 (10), 685–696. 10.1038/nrg2841 20847746

[B29] Moghbeli M. Forghanifard M. M. Sadrizadeh A. Mozaffari H. M. Golmakani E. Abbaszadegan M. R. (2015). Role of Msi1 and MAML1 in Regulation of Notch Signaling Pathway in Patients with Esophageal Squamous Cell Carcinoma. J. Gastrointest. Canc 46 (4), 365–369. 10.1007/s12029-015-9753-9 26294058

[B30] Murugaesu N. Wilson G. A. Birkbak N. J. Watkins T. B. K. McGranahan N. Kumar S. (2015). Tracking the Genomic Evolution of Esophageal Adenocarcinoma through Neoadjuvant Chemotherapy. Cancer Discov. 5 (8), 821–831. 10.1158/2159-8290.cd-15-0412 26003801 PMC4529488

[B31] Ng S. B. Buckingham K. J. Lee C. Bigham A. W. Tabor H. K. Dent K. M. (2010). Exome Sequencing Identifies the Cause of a Mendelian Disorder. Nat. Genet. 42 (1), 30–35. 10.1038/ng.499 19915526 PMC2847889

[B32] Pennathur A. Gibson M. K. Jobe B. A. Luketich J. D. (2013). Oesophageal Carcinoma. The Lancet 381 (9864), 400–412. 10.1016/s0140-6736(12)60643-6 23374478

[B33] Pleasance E. D. Cheetham R. K. Stephens P. J. McBride D. J. Humphray S. J. Greenman C. D. (2010). A Comprehensive Catalogue of Somatic Mutations from a Human Cancer Genome. Nature 463, 191–196. 10.1038/nature08658 20016485 PMC3145108

[B34] Roth A. Khattra J. Yap D. Wan A. Laks E. Biele J. (2014). PyClone: Statistical Inference of Clonal Population Structure in Cancer. Nat. Methods 11 (4), 396–398. 10.1038/nmeth.2883 24633410 PMC4864026

[B35] Scheithauer W. (2004). Esophageal Cancer: Chemotherapy as Palliative Therapy. Ann. Oncol. 15 (6), iv97–iv100. 10.1093/annonc/mdh911 15477344

[B36] Song Y. Li L. Ou Y. Gao Z. Li E. Li X. (2014). Identification of Genomic Alterations in Oesophageal Squamous Cell Cancer. Nature 509 (7498), 91–95. 10.1038/nature13176 24670651

[B37] Sottoriva A. Spiteri I. Piccirillo S. G. M. Touloumis A. Collins V. P. Marioni J. C. (2013). Intratumor Heterogeneity in Human Glioblastoma Reflects Cancer Evolutionary Dynamics. Proc. Natl. Acad. Sci. 110 (10), 4009–4014. 10.1073/pnas.1219747110 23412337 PMC3593922

[B38] The Cancer Genome Atlas Network (2012). Comprehensive Molecular Characterization of Human colon and Rectal Cancer. Nature 487 (7407), 330–337. 10.1038/nature11252 22810696 PMC3401966

[B39] The Cancer Genome Atlas Research Network (2017). Integrated Genomic Characterization of Oesophageal Carcinoma. Nature 541 (7636), 169–175. 10.1038/nature20805 28052061 PMC5651175

[B40] Wang A. Li Z. Wang M. Jia S. Chen J. Ji K. (2020). Molecular Characteristics of Synchronous Multiple Gastric Cancer. Theranostics 10 (12), 5489–5500. 10.7150/thno.42814 32373223 PMC7196298

[B41] Xu J. Cai L. Liao B. Zhu W. Yang J. (2020). CMF-Impute: An Accurate Imputation Tool for Single-Cell RNA-Seq Data. Bioinformatics 36 (10), 3139–3147. 10.1093/bioinformatics/btaa109 32073612

[B42] Yachida S. Jones S. Bozic I. Antal T. Leary R. Fu B. (2010). Distant Metastasis Occurs Late during the Genetic Evolution of Pancreatic Cancer. Nature 467 (7319), 1114–1117. 10.1038/nature09515 20981102 PMC3148940

[B43] Zhang L. Zhou Y. Cheng C. Cui H. Cheng L. Kong P. (2015). Genomic Analyses Reveal Mutational Signatures and Frequently Altered Genes in Esophageal Squamous Cell Carcinoma. Am. J. Hum. Genet. 96 (4), 597–611. 10.1016/j.ajhg.2015.02.017 25839328 PMC4385186

[B44] Zhang X. C. Xu C. Mitchell R. M. Zhang B. Zhao D. Li Y. (2013). Tumor Evolution and Intratumor Heterogeneity of an Oropharyngeal Squamous Cell Carcinoma Revealed by Whole-Genome Sequencing. Neoplasia 15 (12), 1371–1378. 10.1593/neo.131400 24403859 PMC3884528

[B45] Zhu Y.-H. Fu L. Chen L. Qin Y.-R. Liu H. Xie F. (2013). Downregulation of the Novel Tumor Suppressor DIRAS1 Predicts Poor Prognosis in Esophageal Squamous Cell Carcinoma. Cancer Res. 73 (7), 2298–2309. 10.1158/0008-5472.can-12-2663 23436800

[B46] Zhuang J. Cui L. Qu T. Ren C. Xu J. Li T. (2021). A Streamlined scRNA-Seq Data Analysis Framework Based on Improved Sparse Subspace Clustering. IEEE Access 9 (99), 9719–9727. 10.1109/access.2021.3049807

